# A simple molecular mechanism explains multiple patterns of cell-size regulation

**DOI:** 10.1371/journal.pone.0182633

**Published:** 2017-08-16

**Authors:** Morgan Delarue, Daniel Weissman, Oskar Hallatschek

**Affiliations:** 1 Departments of Physics and Integrative Biology, University of California, Berkeley, California 94720, United States of America; 2 Institute for Systems Genetics, University of New York Langone Medical Center, New York, United States of America; 3 Department of Physics, Emory University, Atlanta, GA 30322, United States of America; Texas A&M University College Station, UNITED STATES

## Abstract

Increasingly accurate and massive data have recently shed light on the fundamental question of how cells maintain a stable size trajectory as they progress through the cell cycle. Microbes seem to use strategies ranging from a pure sizer, where the end of a given phase is triggered when the cell reaches a critical size, to pure adder, where the cell adds a constant size during a phase. Yet the biological origins of the observed spectrum of behavior remain elusive. We analyze a molecular size-control mechanism, based on experimental data from the yeast *S. cerevisiae*, that gives rise to behaviors smoothly interpolating between adder and sizer. The size-control is obtained from the accumulation of an activator protein that titrates an inhibitor protein. Strikingly, the size-control is composed of two different regimes: for small initial cell size, the size-control is a sizer, whereas for larger initial cell size, it is an imperfect adder, in agreement with recent experiments. Our model thus indicates that the adder and critical size behaviors may just be different dynamical regimes of a single simple biophysical mechanism.

## Introduction

Cells need to coordinate growth and division to keep their size in the physiologically optimal range [1–4]. A variety of mechanistic models for how cells can link division to growth have been proposed [5–14], and numerous molecular studies have uncovered much of the network of genes and reactions involved [15–20]. Recently, high-throughput experimental techniques have enabled the detailed measurement of size dynamics at the level of single cells [21–24], revealing in detail how cells fluctuate around their typical size; these results have been described by phenomenological models ignoring molecular mechanisms [24, 25]. Here we connect the molecular and the phenomenological approaches by showing that a molecular “titration” model of cells’ response to size fluctuations [5, 7, 9, 26] (also known variously as the “concentration”, “inhibitor-dilution”, or “structural” model depending on the interpretation [7]) gives rise to and constrains the observed phenomenological patterns of size control in different genetic backgrounds.

The basic idea of a titration model is that a transition is triggered when the concentration of an activator exceeds a threshold set by the concentration of a repressor or inhibitor. Our framework differs from existing titration models in three key points that are needed to connect it to the observed single-cell size data [16, 27, 28]. First, while the simplest versions of the model assume that the activator concentration is initially negligible and the amount of repressor is constant (see [7], “structural model”) or that their concentrations are in a quasi-steady state [7, 9, 17, 29], we consider their variation across cells and over time. Second, the reaction between the activator and the inhibitor occurs in a subcellular compartment, such as the nucleus or the cell membrane, that does not necessarily scale linearly with cell volume. Third, while many phenomenological models focus on describing the total change in size over one whole cell cycle [7, 25, 30], cells only regulate their size in certain phases of the cell cycle, such as the B and D intervals in *E. coli* [31], or the G1 or G2 phases for the budding yeast *S. cerevisiae* [28]. Thus, we use a titration model to describe the regulation of a single phase of the cell cycle, with the full size regulation being composed of a series of such steps involving different pathways.

We focus on the first phase of the budding yeast *S. cerevisiae* cell cycle, the G1 phase (from birth to bud), for which recent experiments provide detailed information on regulation at the single-cell and molecular levels [28, 32, 33]. In this case, the activator and repressor are, respectively Cln3 and Whi5, which react in the cell nucleus [34, 35]. The nucleus grows in time, scaling with cell volume [36]. (We consider other possible scalings below.) The key to the usefulness of Cln3 and Whi5 in sensing cell size are their different patterns of production and degradation. Cln3 molecules are produced in the cytosol during G1 at a rate proportional to cell volume [33] and degraded at a (fairly rapid) constant rate [37, 38], while Whi5 is neither produced nor effectively degraded during G1 [16], and so remains constant in number. The proteins are transported inside the nucleus. We assume that the transport of proteins is fast, such that they are essentially entirely concentrated in the nucleus.

## Results

We assume that the cell volume *v* grows exponentially at rate *k* with the time *t* since birth [1, 27, 39]. Then if *κ*_*p*_ is the effective rate density at which Cln3 is accumulating in the nucleus per unit volume, and *k*_*d*_ is the rate at which it effectively degrades in the nucleus, the concentration of Cln3 *c*_*a*_(*t*) evolves as [9]:

$$\frac{dc_{a}}{dt}=\frac{1}{v}\frac{dN_{a}}{dt}-\frac{1}{v}\frac{dv}{dt}c_{a}=\kappa_{p}-(k_{d}+k)c_{a}$$ (1)

*N*_*a*_ being the number of activator. Since the inhibitor is constant in number, as the cell grows its concentration *c*_*i*_(*t*) decreases as:

$$\frac{dc_{i}}{dt}=-kc_{i}$$ (2)

We wish to analyze the predictions of this model for the dynamics of cells that are born with varying initial volume, *v*_0_ = *v*(0). To do this, we need to understand the boundary conditions of Eq (1). The initial concentration *c*_*a*,0_ of Cln3 is roughly independent of *v*_0_ [33], consistent with it being close to an equilibrium of Eq (1). In contrast, it is the *absolute number* of Whi5 per cell, rather than its concentration, that is roughly independent of initial cell size, as it is produced at a roughly size-independent rate [33] during the budded phase, whose duration is not dependent on cell size. We denote by *N*_*i*,0_ this constant initial number of inhibitor molecules. How the cell synthesizes Whi5 at a constant rate independent of its size is an interesting question that we will not attempt to answer here. In the “Analysis” Section of the S1 File, we show that the fact that *N*_*i*,0_ does not depend on *v*_0_ is crucial to maintaining size control. The solutions to Eqs (1) and (2) are:

$$\begin{array}{l} c_{a}\left(t\right)=\frac{\kappa_{p}}{kd+k}+\left(c_{a,0}-\frac{\kappa_{p}}{kd+k}\right)e^{-\left(k_{d}+k\right)t}\\ c_{i}\left(t\right)=\frac{N_{i,0}}{v_{0}}e^{-kt} \end{array}$$ (3)

During the period from birth to time *t*, the cell volume has increased from *v*_0_ to *v*(*t*) = *v*_0_ *e*^*kt*^. In Eq (3), the concentrations of activator and inhibitor can be re-expressed in terms of volume *v*:

$$\begin{array}{l} c_{a}\left(v\right)=\frac{\kappa_{p}}{kd+k}+\left(c_{a,0}-\frac{\kappa_{p}}{kd+k}\right){\left(\frac{v_{0}}{v}\right)}^{1+kd/k}\\ c_{i}\left(v\right)=\frac{N_{i,0}}{v} \end{array}$$ (4)

We observe from Eq (4) that the concentration of activator increases to reach a steady-state concentration, whereas the concentration of inhibitor decreases due to dilution.

**Fig 1:**
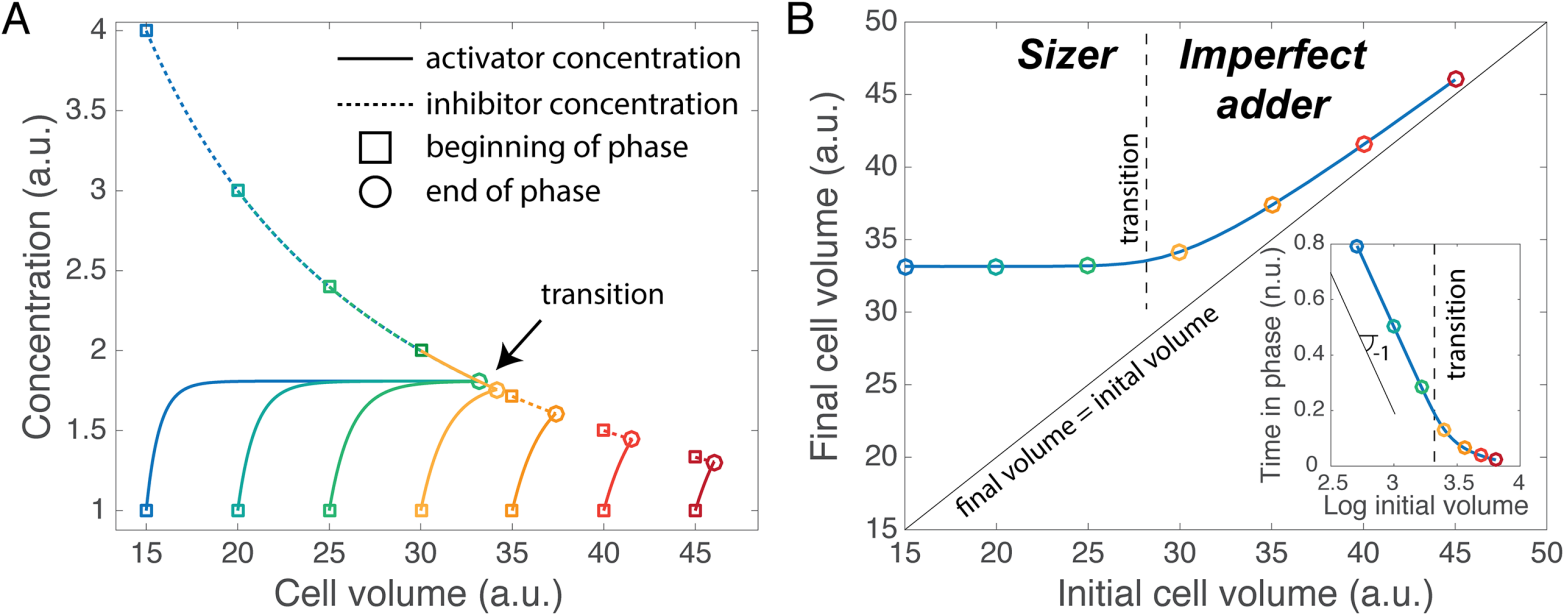
Model. A. Concentration of activator and inhibitor plotted as a function of cell volume for different initial cell volumes, represented by different colors. The beginning of the phase is marked by a square, the end by a circle. B. The final volume, defined when the activator and inhibitor concentrations are equal, is plotted as a function of the logarithm of initial volume. The circles of corresponding color with A are superimposed. Inset: Time spent during the phase (normalized by multiplying by the growth rate) as a function of initial volume. The dashed line separates the two regimes: *sizer* for small initial size, and *imperfect adder* for larger initial size. The parameters are: *κ*_*p*_ = 1.9, *k*_*d*_ = 1.0, *k* = 0.05, *N*_*i*,0_ = 60, *c*_*a*,0_ = 1.

The volume of the cell increases until the activator matches the inhibitor upon which the next phase of the cell cycle is entered, i.e., the final volume *v*_*f*_ satisfies *c*_*a*_ (*v*_*f*_) = *c*_*i*_ (*v*_*f*_). Fig 1A displays the evolution in volume of both activator and inhibitor for different initial cell volume *v*_0_. There are two possible generic behaviors. In the first (blue and green curves in Fig 1), the concentration of activator increases initially (as observed in [33]) to reach a steady-state value that is independent of cell volume, after which the end of the phase is triggered when the inhibitor concentration drops to the same value. In this case, the final volume is independent of the initial volume, i.e., cells are critical sizers. In the second possible behavior (yellow, orange, and red curves in Fig 1), the concentrations of the activator and the inhibitor cross while the former is still increasing. In this case, the final volume does depend on the initial volume.

The exact solution for *v*_*f*_ is unwieldy, but there are two simple limiting regimes that correspond to the qualitative behaviors described above:

$$\begin{array}{c} v_{f}\approx \{\begin{array}{cc} (1+\frac{k_{d}}{k})v^{*}, & \text{if}\;v_{0}\ll v_{T}\\ v^{*}+\chi v_{0}, & \text{if}\;v_{0}\gg v_{T}. \end{array} \end{array}$$ (5)

Here v* = *kN*_*i*,0_/*κ*_*p*_ is the volume scale determined by the basic titration mechanism, *χ* = (1 − (*k* + *k*_*d*_)*c*_*a*,0_/*κ*_*p*_)^*k*/(*k*_*d*_+*k*)^ gives the strength of the dependence of *v*_*f*_ on *v*_0_, and threshold volume separating the regimes is *v*_*T*_ = v*k_*d*_/(*kχ*). Note that because the growth rate *k* is fixed, the cell regulates *v*_*f*_ by controlling the duration *t*_*f*_ of G1, and Eq 5 could equivalently be expressed as an equation for the normalized time *kt*_*f*_ = *ln*(*v*_*f*_/*v*_0_) (Fig 1B, inset).

The first line of Eq (5) shows that for cells that are born small, *v*_0_ ≪ *v*_*T*_, the mechanism enforces a minimum final volume independent of the volume at birth, i.e. a “critical size”, or sizer, as described in [40] for the fission yeast (Fig 1, left side of both panels; note that in the inset, a constant *v*_*f*_ corresponds to a slope of −1 for *kt*_*f*_ vs log *v*_0_). The second line shows that for cells that are born large, *v*_0_ ≫ *v*_*T*_, the final volume is an affine function of the volume at birth. In the limit where the initial activator concentration is small, as in the simple concentration model [7, 29], the slope *χ* = 1 and the mechanism acts as an “adder” (the “incremental” model, [25]). More generally, we define an imperfect adder regime the size-control mechanism when the slope in the second regime is different from 1, *χ* ≠ 1, as in [24]. Note that the threshold size *v*_*T*_ may be very different from the typical size of cells, so that almost all cells in a population may exhibit the same phenomenological pattern of size control (e.g., almost all cells may be “large” in this sense).

Because initial inhibitor concentration decreases with increasing *v*_0_ while initial activator concentration is constant, cells that are born sufficiently large (*v*_0_ > *N*_*i*,0_/*c*_*a*,0_) will have enough activator to trigger the end of G1 immediately upon birth. At this point, our model breaks down and other reactions that are normally rapid compared to the duration of G1 will set the timescale [27]. This breakdown of the model could also explain why mother budding yeast cells, which keep on increasing in birth size generation after generation, do not seem to exhibit any size-control in G1 [27].

**Fig 2:**
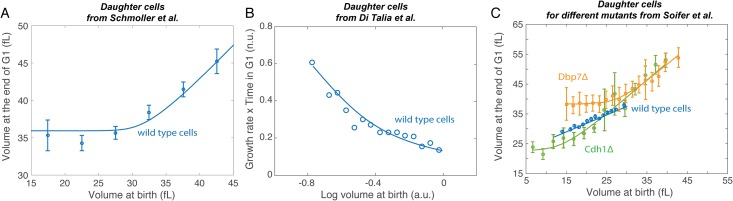
Experimental results. A. Volume at the end of G1 as a function of volume at birth for daughter budding yeast cells. Data from [16], carbon source: ethanol, background isogenic to W303. B. Normalized time spent in G1 as a function of the logarithm of volume at birth for small daughter budding yeast cells. Here, the data from [27] do not show the volume at the end of the phase at a function of the initial cell volume, but another correlation (time spent in the phase as a function of logarithm of volume at birth) between cell cycle parameters, that we are also able to predict. Glucose as carbon source, background isogenic to W303, population enriched in small daughter budding yeast cells following protocol established in [41]. C. Volume at the end of G1 as a function of volume at birth for daughter cells. Data from [28]. Glucose as carbon source, background isogenic to BY4741.

To test our predictions, we reanalyzed data on the budding yeast *S. cerevisiae* from Schmoller et al. [33], Di Talia et al. [27], and Soifer et al. ([28], unpublished data) (Fig 2). Note that, because the growth conditions as well as the strain backgrounds are different, we cannot comment on differences in the absolute value of cell volume between these three independent sets of data. In their study, Schmoller et al. and Di Talia et al. systematically estimated the regulation in size of small daughter cells in wild-type yeast (either smaller due to growth conditions, or enriched in smaller cells following a protocole established in [41]). Our model is consistent with both the Schmoller and the di Talia large and independent datasets, the one from Schmoller et al plotting the volume at G1 as a function of volume at birth [33], and the second one plotting the normalized time spent in G1 as a function of the logarithm of the volume at birth [27], for which we also have a prediction. Both sets of data show the predicted transition between the critical sizer and imperfect adder regimes (Fig 2A and 2B). Soifer et al. also collected data on size regulation in wild-type daughter cells (Fig 2C, blue points), but without enriching for small cells, so the critical sizer regime is not observed.

In addition to the wild type, Soifer et al. systematically measured the volume of cells at the beginning and end of G1 for 520 single-gene knock-out mutants affecting cell size. Of these 520, 490 appear, like the wild-type, to be mostly in the imperfect adder regime, with little sign of the critical sizer regime. The distribution of slopes of *v*_*f*_ vs *v*_0_ is peaked around the wild-type value of *χ* ≈ 0.5 but broadly distributed, with standard deviation *σ*(*χ*) = 0.2 (Fig A in S1 File), indicating that *χ* is not robust to mutation.

The remaining 30 mutants cannot be described by a simple affine relationship between *v*_0_ and *v*_*f*_ (e.g., Fig 2C, Dbp7 and Cdh1 mutants; see Supplementary Information for all 30 mutants). For all 30, we find the pattern predicted by the model: a sizer for small cells crossing over to an imperfect adder for larger ones (Fig B in S1 File). There is no specific gene ontology class that is shared by these 30 mutants (Table A in S1 File). This makes sense, as processes ranging from protein degradation to ribosome biogenesis all affect the dynamics of our model. The average cell size of these mutants is not significantly smaller than wild-type (mean volume normalized to wild-type is 0.96 ± 0.13), and cannot account for the observed behavior (for instance, the Cdh1 and Dbp7 mutants are respectively 8% smaller and 11% larger than wild-type). This indicates that the mutations are instead increasing the transition volume *v*_*T*_ between the regimes so that it lies within the typical size range. The mutants do have unusually small values of the basic volume scale v* and unusually large values of *χ* (the slope of *v*_*f*_ vs. *v*_0_ in the imperfect adder regime), as well as a large ratio of degradation to growth, *k*_*d*_/*k* (Fig A in S1 File). The fact that all these parameters mutate in a coordinated way, as we can observe in Fig A in S1 File, where there is a strong correlation between *χ* and v*, suggests that mutations are canalized to act on only a few parameter combinations while leaving others unchanged. One possibility is that every parameters of the model, which encompass various biological processes, may depend on key physiological parameters such as global protein production rate, which could be affected in every mutations, thus leading to a coordinated variation in different genetic backgrounds.

We have focused on the G1 phase in *S. cerevisiae*, but the titration mechanism of cell size control may be more widespread. In general, however, the activator and inhibitor may be accumulating in a compartment whose volume does not scale with the overall volume of the cell. For instance, it is believed that the fission yeast *S. pombe* divides when a protein, Cdr2, accumulates to a threshold concentration on a region of the cell surface, thus setting a critical surface area, rather than a critical volume [17]. To treat this case, let the volume (or area) of the compartment *v*_*c*_ grow as a power of the cell volume:

$$v_{c}=\epsilon_{s}v^{s}$$ (6)

where *s* ≥ 0 is a scaling exponent, and *ϵ*_*s*_ is a geometrical factor with the appropriate units. Our analysis then carries through, replacing *v* (*v*_0_) by *ϵ*_*s*_ *v*^*s*^ ($\epsilon_{s}v_{0}^{s}$), and the specific growth rate *k* by *sk*. The two regimes calculated in Eq (5) become:

$$\begin{array}{c} v_{f}^{s}\approx \{\begin{array}{cc} (1+\frac{k_{d}}{k})v^{*s}, & \text{if}\;v_{0}^{s}\ll v_{T}^{s}\\ v^{*s}+\chi_{s}v_{0}^{s}, & \text{if}\;v_{0}^{s}\gg v_{T}^{s}. \end{array} \end{array}$$ (7)

with v*^s^ = *skN*_*i*,0_/*ϵ*_*s*_ *κ*_*p*_ the volume scale, *χ*_*s*_ = (1 − (*k* + *sk*_*d*_)*c*_*a*,0_/*κ*_*p*_)^*sk*/(*k*_*d*_ + *sk*)^, and $v_{T}^{s}=v^{*s}k_{d}/\left(sk\chi_{s}\right)$. This regulation breaks down when *s* = 0: in this particular case, the inhibitor is never diluted, and its concentration remains *c*_*i*_(*v*) = *N*_*i*,0_/*ϵ*_0_. Hence, from Eq (3), we observe that the end of the phase is triggered at a time *t*_*f*_ independent of initial volume:

$$t_{f}=\frac{1}{k_{d}}\text{ln}(\frac{1-N_{a,0}k_{d}\epsilon_{0}/\kappa_{p}}{1-N_{i,0}k_{d}\epsilon_{0}/\kappa_{p}})$$ (8)

The size control is lost, and this phase is a timer.

We also treated the case of linear growth for a compartment that does not scale linearly with cell volume (see “Linear growth case” Section of the S1 File). Interestingly, only the sizer regime is present if cell volume grows linearly in time, and the final volume smoothly transits to the loss of size control (*v*_*f*_ = *v*_0_). However, in both exponential and linear growth cases, during the sizer regime, the number of activator grows as *v*^*s*^, and the end of the phase is triggered at a constant $v_{f}^{s}$. These results are in perfect agreement with the case of *S. pombe*, where the authors found that cell division is triggered at a critical surface, and that the activator Cdr2 accumulates proportionally to cell surface (case *s* = 2/3) [17].

## Discussion

In this letter, we analyze the titration model of size control, in which a phase of the cell cycle ends when the concentration of an activator protein exceeds a threshold set by a repressor protein. We have only considered the deterministic case of a simple one-to-one correspondence between the two proteins: *c*_*a*_(*v*_*f*_) = *c*_*i*_(*v*_*f*_). More generally, the actual correspondence between the activator and inhibitor concentration that would set the end of the phase can be more complicated, in the form of a non-linear function *c*_*a*_(*v*_*f*_) = *f* (*c*_*i*_(*v*_*f*_)). This could for instance be the case of non-stochiometric reactions, where multiple interaction events between activators and one inhibitor could occur. We find that as long as the initial number of inhibitor is constant, size control is maintained, regardless of the functional form of the initial amount of activator. In particular, in the case where *N*_*i*,0_ = constant, and *c*_*a*,0_ = constant, the 2 regimes that we found in the manuscript still hold (details of the calculation in the “Analysis” Section of S1 File).

Given that these conditions are met, we predict that any single genotype will show two different patterns of size control in a titration class of models, depending on the initial cell volume: a critical size for small initial volumes, and an imperfect adder for large initial volumes. We find this two-stage pattern in many mutant strains of budding yeast, as well as in wild type situations for small(er) daughter cells. All other observed mutants are consistent with the model, in that they follow the imperfect adder pattern. Study at the population level seem to agree with this description as well, where it has been observed that very large mutants seem to posses a weaker size control than smaller mutants [42]. We predict that for these mutants, careful measurement of rare small cells would reveal the sizer regime; such measurements may be practical with microfluidic techniques [43]. These techniques could also enable the observation of the anticipated breakdown of the model for rare extremely large cells [23].

While we have focused on budding yeast, the model is generic and does not rely on the molecular details of the full cell cycle pathway; even in budding yeast, the activator and repressor in the model may be effective quantities that do not correspond exactly to Cln3 or Whi5. Indeed, nothing in the model requires that the “repressor protein” even be a protein; it could just as well be any set of physical sites whose number does not scale linearly with cell volume [7], such as locations on the cell membrane to which the activator needs to bind to trigger division [17]. Thus, we predict that the same patterns of size regulation will also be found in other species that rely on titration for size control.

It has recently been shown in the case of the fission yeast *S. pombe* that the onset of division seems to be triggered at a critical surface, rather than at a critical volume [17], which can be explained within our model through a different scaling of the compartment with cell size. Moreover, the initiation of DNA synthesis in *E. coli* occurs at a critical cell size, even though the cell seems to operate an adder-type size control over the whole cell cycle [18], similar to the “tandem model” suggested by Tyson [44]. The cell cycle of *E. coli* is complex for fast dividing cells, as multiple rounds of DNA replication happen within the same cell. However, Wallden et al. explain the apparent adder at the whole cell cycle level by different size-regulations of different phases of the cell cycle: a critical size control at the onset of DNA synthesis, then a timer [18].

Recently, [30] have found that budding yeast also seems to act as an adder over the whole cell cycle [30]. They explain this behavior through an extended version of the incremental model, with the key hypothesis that the amount of Whi5 present at birth depends on the cell volume at birth, in contradiction with results from Schmoller et al [33] (see discussion in the “Model of budding yeast cell size regulation over the whole cell cycle” Section of S1 File). Another potential explanation for why cells behave as an adder over their whole cell cycle, similar to the one proposed for *E. coli* by Wallden and colleagues [18], is that different phases of the cell cycle have different size-control mechanisms. In particular, the size control could be composed of a size-regulated phase in G1, as explored in this letter, followed by a timer (Fig C in S1 File).

## Supporting information

S1 File Supplementary information. The Supplementary Information contains Supplementary Figs A, B and C, as well as Supplementary Table A. It also contains details of the calculation.
(PDF)
