# Supplementary material for: A simple molecular mechanism explains multiple patterns of cell-size regulation
Source: PLoS One. 2017 Aug 16;12(8):e0182633. doi: 10.1371/journal.pone.0182633 (PMC5558972; doi:10.1371/journal.pone.0182633)
Supplement: S1 File — The Supplementary Information contains Supplementary Figs A, B and C, as well as Supplementary Table A. It also contains details of the calculation. (PDF) [file pone.0182633.s001.pdf]

A simple molecular mechanism explains multiple patterns of cell-size regulation: Supplementary Information

MD, DW, OH

July 28, 2017

Analysis

We present in the following the results in the case where the volume of the compartment scales linearly with cell volume ($s = 1$). However, the conclusions drawn hereafter can be extended to any scaling ($s > 0$).

Solving the dynamical Eq. (4) for the activator concentration c_a as a function of volume v and equating it to a function of the repressor concentration c_i gives an implicit equation for the volume v_f at the end of the cell cycle phase: $c_a(v_f) = f\left(c_i(v_f)\right)$. In the manuscript, we considered the cases where (i) f is the unity function, and (ii) $N_{i,0}$ is a constant. In the following, we generalize our study to any functional form of f, and explore different possibilities of initial conditions. Eq. (4) can be re-expressed as follow:

$$1 - \left(1 - \frac{c_{a,0}(k_d + k)}{\kappa_p}\right)\left(\frac{v_0}{v_f}\right)^{1+k_d/k} = \frac{k_d + k}{\kappa_p} f\left(\frac{N_{i,0}}{v_f}\right) \tag{S1}$$

Let's explore the conditions that are necessary to enforce size-control. Size-control is lost when the final volume is proportional to the initial volume: $v_f = \beta v_0$ where $\beta = e^{kt_f}$ for exponential growth (case of a timer). In this condition, the left-hand side of Eq. (S1) is independent of v_0 if $c_{a,0}$ is a constant. Assuming that there is an inhibitor (that is f is not a constant), and expressing $N_{i,0} = c_{i,0} v_0$:

$$1 - \left(1 - \frac{c_{a,0}(k_d + k)}{\kappa_p}\right)\beta^{-(1+k_d/k)} = \frac{k_d + k}{\kappa_p} f\left(\frac{c_{i,0}}{\beta}\right) \tag{S2}$$

We conclude from the expression of Eq. (S2) that two different conditions can lead to a loss of size control: (i) the inhibitor and activators are regulated in such a way that their respective initial concentrations satisfy the condition expressed in Eq. (S2), which is an unlikely scenario, or (ii) both initial concentrations are constant. In this case, Eq. (S2) defines the time t_f at the end of the phase.

Note that if we consider the case where the initial number of activator is held constant, $c_{a,0} = N_{a,0}/v_0$, the previous equation reduces to:

$$1 - \left(1 - \frac{N_{a,0}(k_d + k)}{v_0 \kappa_p}\right)\beta^{-(1+k_d/k)} = \frac{k_d + k}{\kappa_p} f\left(\frac{c_{i,0}}{\beta}\right) \tag{S3}$$

We observe that size control is enforced for small volume (β depends on v_0), but that this scenario breaks down for larger initial volume. Hence, a strict condition that enforces size-control is set at the inhibitor level: size-control is lost as soon as its initial concentration is constant.
Assuming this is not the case, and that its initial number is constant, then size-control is enforced, whichever the relationship between the final concentrations of activator and inhibitor. In particular, both the initial critical size regime and the imperfect adder regime are still holding. Indeed, for $v_f \gg v_0$, Eq. (S1) reduces to:

$$1 = \frac{k_d + k}{\kappa_p} f\left(\frac{N_{i,0}}{v_f}\right) \tag{S4}$$

which leads to:

$$v_f = \frac{N_{i,0}}{f^{-1}\left(\frac{\kappa_p}{k_d+k}\right)} \tag{S5}$$

with f^{-1} being the inverse function of f. For large initial volume, and Taylor expanding f to first order ($f(x) = \alpha_0 + \alpha_1 x$), Eq. (S1) reduces to:

$$v_f = \alpha_1 v^* + (\chi + \alpha_0)\, v_0 \tag{S6}$$

Hence, in general, there can be size control as long as $c_{i,0} \neq 0$. For instance, there can be a case where $N_{i,0} = a + bv_0$ that will still enforce size control. However, in this case, both regimes will be affine functions of initial volume.

Linear growth case

Some organisms, like the fission yeast *S. pombe*, are believed to display a linear growth, based on the measurement of the cell's length over time (1). The starting point for the linear growth case is the same as for the exponential growth, but now $\partial_t v = \alpha$ for some constant α. The decrease in inhibitor concentration remains unchanged. However, the equation governing the dynamics of activator changes:

$$\frac{dc_a}{dt} = \kappa_p - k_d - c_a \frac{\alpha}{v} c_a \tag{S7}$$

The resolution of this equation yields, replacing time by volume, like in Eq. (4):

$$c_a(v) = \left(c_{a,0} v_0 - \frac{\kappa_p}{k_d}\left(v_0 - \frac{\alpha}{k_d}\right)\right) \frac{1}{v} e^{-k_d(v-v_0)/\alpha} + \frac{\kappa_p}{k_d v}\left(v - \frac{\alpha}{k_d}\right) \tag{S8}$$

The implicit solution for the final volume becomes:

$$\left(c_{a,0} v_0 - \frac{\kappa_p}{k_d}\left(v_0 - \alpha/k_d\right)\right) e^{-(v_f - v_0)k_d/\alpha} = N_{i,0} - \frac{\kappa_p}{k_d}\left(v_f - \alpha/k_d\right). \tag{S9}$$

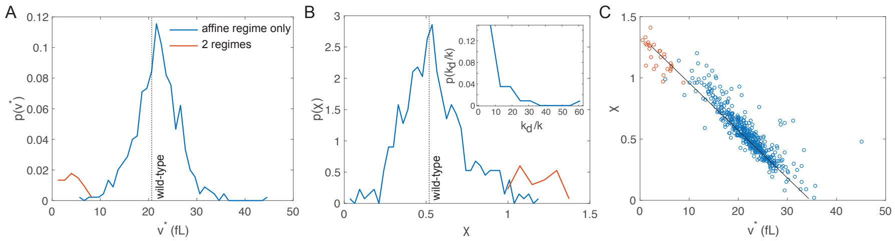

Figure A: Results of the fitting of data from Soifer et al. A-B. Probability distribution of the typical volume v^* (A) and slope χ (B), for the 490 one regime mutants (blue) and the 30 two regimes mutants (orange). Inset: histogram of k_d/k obtained from the fit of the 30 mutants. This parameter cannot be obtained for the other 490 mutants. C. Slope χ plotted as a function of typical volume v^* for all mutants.

For $N_{i,0}$ and $c_{a,0}$ independent of v_0, this has the asymptotic solutions:

$$v_f \approx \begin{cases} \alpha/k_d + k_d N_{i,0}/\kappa_p & \text{if } v_0 \ll \alpha/k_d + k_d N_r/\kappa_p \\ v_0 & \text{if } v_0 \gg \alpha/k_d + k_d N_r/\kappa_p. \end{cases} \tag{S10}$$

We see that for linear growth, size-control is enforced for small initial size, whereas it it breaks down for large cells. This is in contrast with the the exponential growth case, where 2 regimes are observed before the break down of the model. The generalization to non-linear scaling of the compartment can be performed in the same way as in the case of exponential growth for small initial volume. We then find that, in particular, if the scaling is not linear, the critical size regime would depend on scaling: For $s = 2/3$, the first regime becomes a critical surface.

S. cerevisiae mutants from Soifer et al.

Out of the 520 budding yeast mutant strains analyzed, the 490 mutant strains of *S. cerevisiae* that are fit well by a single imperfect adder model show a broad distribution of v^* and slopes χ (figure S1A-B), peaked at the wild-type value. 30 mutants did not appear by eye to follow a simple affine pattern. These are shown in Fig. B, and there gene description is presented in Table S1. Note that these 30 mutants are not particularly small as compared to wild-type (see inset value of mean cell volume at birth, normalized to the wild-type value). All 30 matched the predicted pattern of a constant regime followed by an affine regime. The data presented in the main text correspond to the Cdh1 and Dbp7 mutants. We superimposed the distributions of the parameters χ and v^* obtained for these 30 mutants. We observe that they present rather large slope χ and small typical volume v^* as compared to wild type. The variation of these 2 parameters would impose a reduction of the volume at transition, if the ratio k_d/k would not increase. The third parameter of the fit for the 30 mutants is this ratio, and we observe that it indeed increase, up to very large values. We cannot extract this parameter for the other 490 mutants, but can give a lower bound of less than 10.

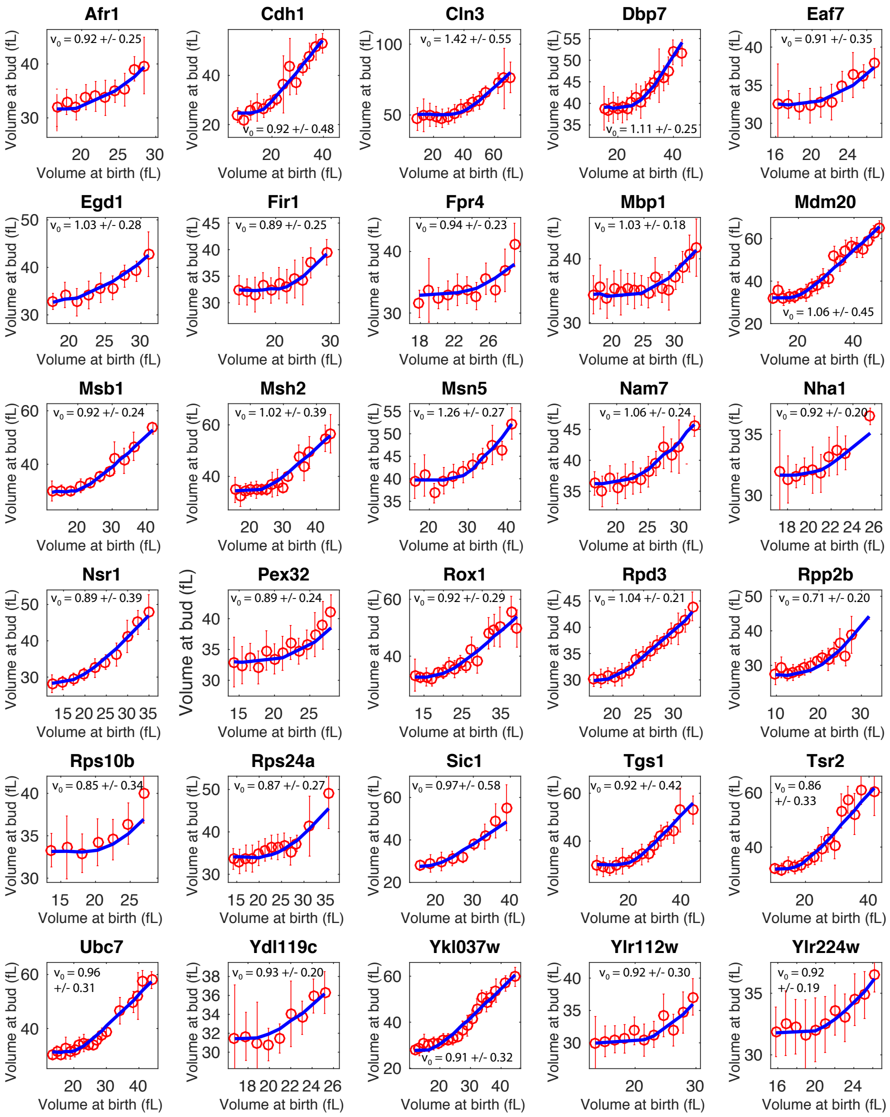

Figure B: Fitting of the 30 mutants displaying the two regimes predicted by our model. Each point represents mean ± SD of the binned data. Each bin contains at least 20 points. The number in the inset represents the mean volume at birth of the mutant, normalized by the mean volume at birth of the wild-type.

Figure S1C displays the slope χ plotted as a function of typical volume v^*. We observe that there seems to be an affine relationship between the two parameters, for all the mutants (one regimes and two regimes alike), suggesting that (i) there must be some sort of compensatory mechanism in the way these two parameters change with a mutation, for which the origin goes beyond the scope of this study, and that (ii) compensatory mechanism is consistent for the apparently 2 different sets of mutants.
Case of the Cln3 mutant:
The fact that the Cln3 knockout still controls its size does indicate the existence of compensatory mechanisms. Our model does not necessitate the absolute presence of Cln3 as activator. Rather, it suggests that Cln3 is a preferred activator, but when deleted, other cyclins can compensate for its loss. This is in agreement with the fact that the slope of the second regime in Cln3Δ is ~ 1, consistent with the absence of initial activator ($c_{a,0} \sim 0$).

Model of the budding yeast cell size regulation over the whole cell cycle.

We consider recent experiments by I. Soifer and colleagues showing that budding yeast are adders over their whole cell cycle (2), even though the size-control in G1 is an imperfect adder (3). We find that our model can give rise to an adder *over the whole cell cycle* by considering that different phases of the cell cycle have different size-control mechanisms. In contrast to G1, the phase between the end of G1 and division is a "timer" (2–4): its duration, of about $t^* = 50$ minutes, is largely independent of cell size at the end of G1. In this case, the volume at division v_d is simply proportional to the volume at the end of G1 v_{G1}:

$$v_d = v_{G1} e^{kt^*} = (\chi v_0 + v^*) e^{kt^*} \tag{S11}$$

Using the independently-measured parameter values $t^* = 50$ minutes, $k = 0.0096$ min^{-1} (5), Eq. (S11) is in excellent agreement with the experimental data (2) (Fig. S3). The fact that two independent size control mechanisms, the imperfect adder in G1 and the timer following G1, have been tuned to combine to give a near-perfect adder ($\chi e^{kt^*} = 0.92$) suggests that there has been selection for the adder phenotype at the whole cell-cycle level.
Note on the model presented by Soifer et al.:
Soifer et al. show that the adder phenotype could be caused by cells producing a fixed amount of Whi5 in the budded phase (2), and the end of the subsequent G1 phase triggered at a volume proportional to the initial amount of Whi5.
Their model does not agree with experimental results for the following reasons:

1. The authors find within their model that the amount of Whi5 at birth is a function of the cell volume at birth. This is in contradiction with recent results by Schmoller et al. (4).

2. The authors consider that the concentration of Cln3 is constant. This leads to a prediction that the size-control in G1 can only be an imperfect adder, with a slope $\chi \leq 1$. This is in contradiction with three results: the concentration of Cln3 does

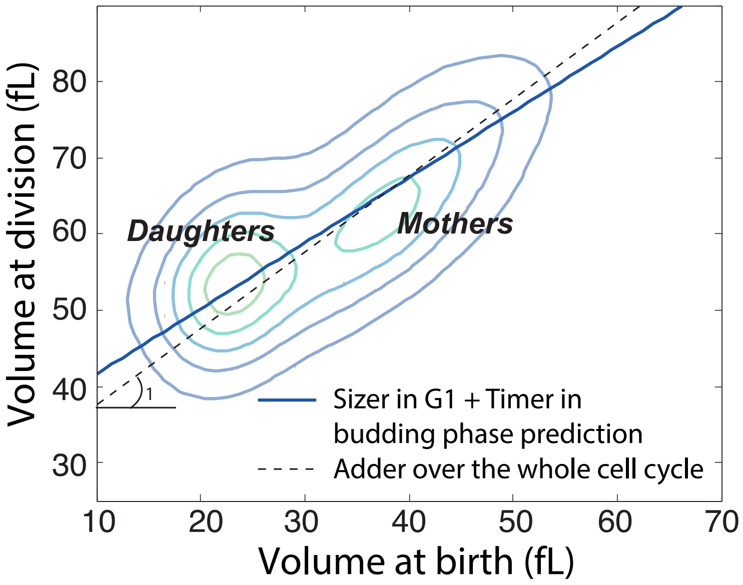

Figure C: Prediction of size-control over the whole cell cycle, where the regulation is composed of a sizer in G1 followed by a timer. The experimental data are from Soifer et al (2), and plotted as a density contour plot. The 2 observed subpopulations correspond to the daughter cell subpopulation, and the mother cell subpopulation. Superimposed is the best fit for an adder regulation (slope of 1 in the plot) that would emerge from the incremental model.

increase at the beginning of G1 to reach a steady-state (4), (ii) we do observe in a number of circumstances the presence of 2 regimes, even in the wild-type situation (Fig. 2), and (iii) in the case that the size-control is only an imperfect adder, we observe instances where the slope $\chi \geq 1$.

Hence, we argue that some of their key hypothesis are not supported experimentally, and lead to predictions that are not verified experimentally, suggesting that the extension of the incremental model, proposed to describe size-control in bacteria and even if it agrees experimentally with some data, still presents some caveats. Our model better takes into account (i) the initial conditions (ii) and the molecular dynamics giving rise to size-control. We find that the pseudo-adder behavior observed over the whole cell cycle may just be the result of different size-control mechanisms in different phases of the cell cycle. With this in mind, we predict that the extended incremental model will break down in the case of certain mutants over the whole cell cycle, as it cannot readily explain the size-regulation of mutants in the G1 phase of the cell cycle.

Name	ORF name	Gene description
Afr1	YDR085c	Protein required for pheromone-induced projection (shmoo) formation; regulates septin architecture during mating; has an RVXF motif that mediates targeting of Glc7p to mating projections; interacts with Cdc12p; AFR1 has a paralog, YER158C, that arose from the whole genome duplication
Cdh1	YGL003c	Activator of anaphase-promoting complex/cyclosome (APC/C); antagonist of the spindle assembly checkpoint; directs ubiquitination of cyclins resulting in mitotic exit; targets the APC/C to specific substrates including: Cdc20p, Ase1p, Cin8p, Fin1p and Clb5p; partially active in metaphase, and fully active in anaphase; cell-cycle regulated
Cln3	YAL040c	G1 cyclin involved in cell cycle progression; activates Cdc28p kinase to promote G1 to S phase transition; plays a role in regulating transcription of other G1 cyclins, CLN1 and CLN2; regulated by phosphorylation and proteolysis; acetyl-CoA induces CLN3 transcription in response to nutrient repletion to promote cell-cycle entry; cell cycle arrest phenotype of the cln1 cln2 cln3 triple null mutant is complemented by any of human cyclins CCNA2, CCNB1, CCNC, CCND1, or CCNE1
Dbp7	YKR024c	Putative ATP-dependent RNA helicase of the DEAD-box family; involved in ribosomal biogenesis; required at post-transcriptional step for efficient retrotransposition; essential for growth under anaerobic conditions
Eaf7	YNL136w	Subunit of the NuA4 histone acetyltransferase complex; NuA4 acetylates the N-terminal tails of histones H4 and H2A
Egd1	YPL037c	Subunit beta1 of the nascent polypeptide-associated complex (NAC); involved in protein targeting, associated with cytoplasmic ribosomes; enhances DNA binding of the Gal4p activator; homolog of human BTF3b; EGD1 has a paralog, BTT1, that arose from the whole genome duplication
Fir1	YER032w	Protein involved in 3' mRNA processing; interacts with Ref2p; APCC(Cdh1) substrate; potential Cdc28p substrate
Fpr4	YLR449w	Peptidyl-prolyl cis-trans isomerase (PPIase); nuclear proline isomerase; affects expression of multiple genes via its role in nucleosome assembly; catalyzes isomerization of proline residues in histones H3 and H4, which affects lysine methylation of those histones; PPIase domain acts as a transcriptional repressor when tethered to DNA by lexA, and repressor activity is dependent on PPIase activity; contains a nucleoplasmin-like fold and can form pentamers
Mbp1	YDL056w	Transcription factor; involved in regulation of cell cycle progression from G1 to S phase, forms a complex with Swi6p that binds to MluI cell cycle box regulatory element in promoters of DNA synthesis genes
Mdm20	YOL076w	Non-catalytic subunit of the NatB N-terminal acetyltransferase; NatB catalyzes N-acetylation of proteins with specific N-terminal sequences; involved in mitochondrial inheritance and actin assembly

Msb1	YOR188w	Protein of unknown function; may be involved in positive regulation of 1,3-beta-glucan synthesis and the Pkc1p-MAPK pathway; multicopy suppressor of temperature-sensitive mutations in CDC24 and CDC42, and of mutations in BEM4; potential Cdc28p substrate; relocalizes from bud neck to cytoplasm upon DNA replication stress
Msh2	YOL090w	Protein that binds to DNA mismatches; forms heterodimers with Msh3p and Msh6p that bind to DNA mismatches to initiate the mismatch repair process; contains a Walker ATP-binding motif required for repair activity and involved in interstrand cross-link repair; Msh2p-Msh6p binds to and hydrolyzes ATP
Msn5	YDR335w	Karyopherin; involved in nuclear import and export of proteins, including import of replication protein A and export of Far1p and transcription factors Swi5p, Swi6p, Msn2p, and Pho4p; required for re-export of mature tRNAs after their retrograde import from the cytoplasm; exportin-5 homolog
Nam7	YMR085c	ATP-dependent RNA helicase of the SFI superfamily; involved in nonsense mediated mRNA decay; required for efficient translation termination at nonsense codons and targeting of NMD substrates to P-bodies; binds to the small ribosomal subunit via an interaction with Rps26; forms cytoplasmic foci upon DNA replication stress
Nha1	YLR138w	Na+/H+ antiporter; involved in sodium and potassium efflux through the plasma membrane; required for alkali cation tolerance at acidic pH
Nsr1	YGR159c	Nucleolar protein that binds nuclear localization sequences; required for pre-rRNA processing and ribosome biogenesis; binds to single stranded telomeric DNA and mRNA; methylated by Hmt1p; interaction with Top1p and nucleolar localization are negatively regulated by polyphosphorylation
Pex32	YBR168w	Peroxisomal integral membrane protein; involved in negative regulation of peroxisome size; partially functionally redundant with Pex31p; genetic interactions suggest action at a step downstream of steps mediated by Pex28p and Pex29p
Rox1	YPR065w	Heme-dependent repressor of hypoxic genes; mediates aerobic transcriptional repression of hypoxia induced genes such as COX5b and CYC7; repressor function regulated through decreased promoter occupancy in response to oxidative stress; contains an HMG domain that is responsible for DNA bending activity; involved in the hyperosmotic stress resistance
Rpd3	YNL330c	Histone deacetylase, component of both the Rpd3S and Rpd3L complexes; regulates transcription, silencing, autophagy and other processes by influencing chromatin remodeling; forms at least two different complexes which have distinct functions and members; Rpd3(L) recruitment to the subtelomeric region is regulated by interaction with the arginine methyltransferase, Hmt1p
Rpp2b	YDR282w	Ribosomal protein P2 beta; a component of the ribosomal stalk, which is involved in the interaction between translational elongation factors and the ribosome; free (non-ribosomal) P2 stimulates the phosphorylation of the eIF2 alpha subunit (Sui2p) by Gcn2p; regulates the accumulation of P1 (Rpp1Ap and Rpp1Bp) in the cytoplasm

Rps10	YOR293w	Protein component of the small (40S) ribosomal subunit; homologous to mammalian ribosomal protein S10, no bacterial homolog; RPS10A has a paralog, RPS10B, that arose from the whole genome duplication; mutations in the human homolog associated with Diamond-Blackfan anemia
Rps24a	YER074w	Protein component of the small (40S) ribosomal subunit; homologous to mammalian ribosomal protein S24, no bacterial homolog; RPS24A has a paralog, RPS24B, that arose from the whole genome duplication
Sic1	YLR079w	Cyclin-dependent kinase inhibitor (CKI); inhibitor of Cdc28-Clb kinase complexes that controls G1/S phase transition, preventing premature S phase and ensuring genomic integrity; phosphorylated by Clb5/6-Cdk1 and Cln1/2-Cdk1 kinase which regulate timing of Sic1p degradation; phosphorylation targets Sic1p for SCF(CDC4)-dependent turnover; functional homolog of mammalian Kip1
Tgs1	YPL157w	Trimethyl guanosine synthase, conserved nucleolar methyl transferase; converts the m(7)G cap structure of snRNAs, snoRNAs, and telomerase TLC1 RNA to m(2,2,7)G; also required for nucleolar assembly and splicing of meiotic pre-mRNAs; interacts with Swm2p, which may confer substrate specificity on Tgs1p
Tsr2	YLR435w	Protein with a potential role in pre-rRNA processing
Ubc7	YMR022w	Ubiquitin conjugating enzyme; involved in the ER-associated protein degradation (ERAD) pathway and in the inner nuclear membrane-associated degradation (INMAD) pathway; requires Cue1p for recruitment to the ER membrane; proposed to be involved in chromatin assembly
Hem25	YDL119c	Mitochondrial glycine transporter; required for the transport of glycine into mitochondria for initiation of heme biosynthesis, with YMC1 acting as a secondary transporter; homolog of human SLC25A38, a mitochondrial glycine transporter associated with nonsyndromic autosomal recessive congenital sideroblastic anemia; human SLC25A38 can complement the heme deficiency associated with the null mutant; GFP-fusion protein is induced in response to the DNA-damaging agent MMS
Aim26	YKL037w	Protein of unknown function; null mutant is viable and displays elevated frequency of mitochondrial genome loss; null mutation confers sensitivity to tunicamycin and DTT
-	YLR112w	Putative protein of unknown function; conserved across S. cerevisiae strains
Ucc1	YLR224w	F-box protein and component of SCF ubiquitin ligase complexes; involved in ubiquitin-dependent protein catabolism; readily monoubiquitinated in vitro by SCF-Ubc4 complexes; SCF-Ucc1 regulates level of Cit2 citrate synthase protein to maintain citrate homeostasis, acts as metabolic switch for glyoxylate cycle.

Table A: Gene description of the 30 mutants displaying the 2 regimes.

References

1. Nobs, J.-B., and S. J. Maerkl, 2014. Long-term single cell analysis of S. pombe on a microfluidic microchemostat array. *PloS ONE* 9:e93466.

2. Soifer, I., L. Robert, and A. Amir, 2016. Single-cell analysis of growth in budding yeast and bacteria reveals a common size regulation strategy. *Current Biology* 26:356–361.

3. Soifer, I., and N. Barkai, 2014. Systematic identification of cell size regulators in budding yeast. *Molecular systems biology* 10:761.

4. Schmoller, K. M., J. Turner, M. Kõivomägi, and J. M. Skotheim, 2015. Dilution of the cell cycle inhibitor Whi5 controls budding-yeast cell size. *Nature* 526:268–272.

5. Ferrezuelo, F., N. Colomina, A. Palmisano, E. Garí, C. Gallego, A. Csikász-Nagy, and M. Aldea, 2012. The critical size is set at a single-cell level by growth rate to attain homeostasis and adaptation. *Nature communications* 3:1012.
